# The role of recombination, niche‐specific gene pools and flexible genomes in the ecological speciation of bacteria

**DOI:** 10.1002/ece3.5052

**Published:** 2019-04-04

**Authors:** Michael Schmutzer, Timothy Giles Barraclough

**Affiliations:** ^1^ Department of Life Sciences Imperial College London Ascot UK

**Keywords:** bacteria, ecological divergence, homologous recombination, speciation

## Abstract

Bacteria diversify into genetic clusters analogous to those observed in sexual eukaryotes, but the definition of bacterial species is an ongoing problem. Recent work has focused on adaptation to distinct ecological niches as the main driver of clustering, but there remains debate about the role of recombination in that process. One view is that homologous recombination occurs too rarely for gene flow to constrain divergent selection. Another view is that homologous recombination is frequent enough in many bacterial populations that barriers to gene flow are needed to permit divergence. Niche‐specific gene pools have been proposed as a general mechanism to limit gene flow. We use theoretical models to evaluate additional hypotheses that evolving genetic architecture, specifically the effect sizes of genes and gene gain and loss, can limit gene flow between diverging populations. Our model predicts that (a) in the presence of gene flow and recombination, ecological divergence is concentrated in few loci of large effect and (b) high rates of gene flow plus recombination promote gene loss and favor the evolution of niche‐specific genes. The results show that changing genetic architecture and gene loss can facilitate ecological divergence, even without niche‐specific gene pools. We discuss these results in the context of recent studies of sympatric divergence in microbes.

## 1 INTRODUCTION

Bacteria and Archaea together comprise at least 10^6^ species (Louca, Mazel, Doebeli, & Wgener Parfrey, 2019) and, by some accounts, as many as 10^10^ species (Bell, 2009). Yet, despite their ubiquity, abundance and diversity, the nature of bacterial diversity is a long‐standing problem (Cohan & Perry, 2007). Species are often assumed to exist (Doolittle, 2008) and indeed surveys of DNA sequence variation confirm the existence of genetic clusters equivalent to those observed in eukaryotes (Acinas, Klepac‐Ceraj, Hunt, & Pharino, 2004; Polz, Alm, & Hanage, 2013). Statistical analyses of large samples show that these clusters indicate independently evolving sets of individuals rather than chance products of neutral coalescence (Barraclough, Hughes, Ashford‐Hodges, & Fujisawa, 2009). Nevertheless, the variety of mechanisms of recombination in bacteria, which ranges from absent (i.e., strictly clonal) to highly recombining via several processes, has confounded development of a standard concept for bacterial species and speciation equivalent to the biological species concept in sexual eukaryotes. In practice, many researchers are agnostic about whether species even exist in bacteria and use simple genetic thresholds or criteria for classifying species (Carporaso et al., 2010). This approach, however, does not help to uncover the evolutionary causes of diversification.

Recently, there have been growing efforts to frame species and speciation of Bacteria and Archaea in population genetic theory equivalent to theories for sexual eukaryotes. A major topic is whether recombination and gene flow between diverging populations plays an important role in bacterial speciation. Emphasizing the importance of ecological adaptation and clonal reproduction, Cohan and co‐workers propose that adaptation to distinct ecological niches is the principal cause of bacterial species (which they call ecotypes). Periodic selective sweeps reduce variation across the whole genome of a clonal niche‐specialist while driving the accumulation of divergence between ecotypes (Cohan and Perry 2007), leading to a pattern of discrete genetic clusters. Recombination rates are proposed to be too low to produce maladapted “hybrid” genotypes and erode differences between ecotypes, and therefore no special mechanisms are needed to prevent gene flow between diverging populations.

In contrast, other authors emphasize the role of recombination in bacterial divergence (Fraser, Alm, Polz, Spratt, & Hanage, 2009; Hanage, Fraser, and Spratt, 2006; Retchless & Lawrence, 2007). Bacteria reproduce clonally but recombination occurs via several mechanisms that are decoupled from reproduction. Homologous recombination (HR) involves “copy and paste” of DNA from the environment that requires tracts of similar sequence at either end of the pasted DNA. Rates vary widely by several orders or magnitude, up to more than 10× the per nucleotide effect of mutation (Vos, Hesselman, Beek, Passel, & Eyre‐Walker, 2015), which is sufficient to constrain multilocus divergence under neutral conditions (Fraser et al. 2009). Furthermore, several genomic studies report gene‐specific sweeps during ecological divergence, which are only possible with recombination (Cordero and Polz, 2014; Shapiro et al. 2012), rejecting the ecotype model of divergence with strictly clonal evolution. Akin to theories of speciation in sexual eukaryotes, it has been proposed therefore that recombination rates and gene flow between populations might be high enough in some bacteria to constrain multilocus divergence. Consequently, ecological speciation might require mechanisms to prevent gene transfer, analogous to reproductive isolating mechanisms in plants and animals (Polz et al. 2013).

Several mechanisms have been proposed for restricting HR between bacterial species. First, rates of HR decline as sequence divergence increases, which could act as a barrier to gene flow between populations (Fraser et al. 2009). This has been dismissed as a general explanation because HR depends less stringently on genetic similarity in Archaea (Polz et al. 2013), which nonetheless show similar patterns of genetic clustering (Cadillo‐Quiroz et al., 2012). Second, there might be specific mechanisms to reduce gene exchange, for example, incompatible pheromone systems (Carrolo, Pinto, Melo‐Cristino, & Ramirez, 2009) or incompatible restriction enzymes (Jeltsch, 2003). While reported in some bacteria (e.g., *Streptococcus*), the general prevalence of such isolating mechanisms remains unclear.

A popular recent hypothesis is that restriction of gene transfer within habitat patches might restrict gene flow and permit ecological divergence, if populations adapting to distinct niches are only exposed to gene pools in their local habitat patch rather than globally across habitat types (Polz et al. 2013). This mechanism is analogous to habitat‐based assortative mating in models of eukaryote speciation (Diehl & Bush, 1989). It relies on general features of the environment rather than specific genetic mechanisms and, hence, applies broadly across Bacteria and Archaea. It is also consistent with observed reductions of HR between ecologically distinct species (Polz et al. 2013). Melendrez et al. (2016) also found that gene‐specific selective sweeps occur within ecologically divergent species of *Synechococcus*, but in rebuttal of this hypothesis, they argued that separate gene pools were a consequence of ecological divergence rather than a cause. In theory, strong enough selection against recombinant genotypes could permit divergence even without niche‐specific gene flow at the outset (Bürger, 2014; Gavrilets, 2004).

Resolving the role of recombination in bacterial speciation, and of niche‐specific gene pools, in particular, depends critically on genetic architecture. Modelling of ecological divergence with gene flow in sexually reproducing organisms shows that gene flow has the greatest inhibitory effect on divergence when divergence involves many genes of small effects rather than few genes of large effect (Gavrilets, 2004; Kondrashov, 1986). With many genes of small effects, niche‐specific assortative mating greatly enhances the progress of divergence (Diehl & Bush, 1989; Felsenstein, 1981). Friedman, Alm, and Shapiro (2013) extended this work to clonal bacteria with varying rates of HR. They showed that the constraining effect of HR between diverging populations increased with the number of loci, as in sexual models. They proposed reducing the number of loci needed for speciation, as well as niche‐specific gene pools, as possible mechanisms to overcome this, without modelling them explicitly, and therefore, we construct models to explore those processes here.

We use simulations of a mathematical model to investigate the interplay between genetic architecture and niche‐specific gene pools in facilitating ecological divergence of bacteria. Specifically, we ask whether evolving architecture provides an additional mechanism to aid ecological divergence. We investigate two ways in which genetic architecture can evolve; (a) through changes in the effects of loci on a trait affecting fitness, and (b) through changes in the number of loci through gene gain and loss. Previous models of sexual eukaryotes showed that evolving effect sizes of loci can protect locally adapted genotypes from the homogenizing effects of gene flow because the genetic basis of ecological traits becomes concentrated into fewer genes of large effect (Yeaman & Whitlock, 2011). We further evaluate this idea to the case of bacteria with HR, which is not completely equivalent to meiotic recombination, as it tests a single maladapted gene region arriving by gene flow in an otherwise fully adapted genome.

A second aspect of genetic architecture that needs to be considered in modelling the effects of recombination on bacterial divergence is gene content. In contrast to eukaryotes, gene gain and loss is ubiquitous in bacteria. Among related strains, this divides the genome into a “flexible” part that differs between strains, and a “core” part that is shared (Cordero and Polz, 2014). The rate of turnover sometimes exceeds thousands of gene gain and loss events per 1% amino acid divergence (Nowell, Green, Laue, & Sharp, 2014). Variation in gene content is often related to ecological traits. For example, within the marine cyanobacterium *Prochlorococcus*, both Atlantic and Pacific populations carried low‐frequency genes as part of their “flexible” genomes, most of which were found on average in only one in four isolates. However, 29 genes associated with phosphorus uptake and metabolism had markedly different frequencies between populations; nearly absent in the Pacific, but close to fixation in the Atlantic (Coleman and Chisholm, 2010).

Clearly, gene gain is important for adaptive novelty and allowing bacteria to adapt to new conditions. Many accounts consider how flexible genomes aid colonization of new habitats, the spread of antibiotic resistance, and adaptation to fluctuating selection (Niehaus, Mitri, Fletcher, & Foster, 2015). Here, we focus instead on the effect of flexible genomes on divergence with gene flow, which has not previously been investigated in models of bacterial or eukaryote divergence. We hypothesize that niche‐specific traits encoded by differences in gene content, that is, nonhomologous loci are protected against gene flow even if gene flow via HR occurs at sufficient rate to homogenize the core genome. Hence, both evolution of the effect sizes of loci and niche‐specific genes might provide further isolating mechanisms against gene flow via HR between locally adapted populations, which we evaluate in models with or without niche‐specific gene pools.

## 2 METHODS

### 2.1 Model description

#### 2.1.1 Motivation

Our model of divergence between two subpopulations occupying distinct habitats but connected by DNA flow is inspired by the study of Shapiro et al. (2012). They reported on population divergence in *Vibrio cyclitrophicus*, a marine bacterium adapting to two niches in sympatry while undergoing high rates of HR. The ecotypes occupy different sized particles in the water column that are found in close proximity. The study found that genetic differentiation between the two forms (called L and S strains) was restricted to 11 discrete genome regions, with >80% of Single Nucleotide Polymorphism (SNPs) found in three regions. In contrast, the rest of the genome was intermingled between the two forms, with overall rates of HR estimated at 1000× mutation rates. One of their three questions for future investigation was “what are the barriers to gene flow between sympatric ecological populations?”, which we investigate here. In order to focus on the effects of shared versus separate gene pools in our model, we assume that cells do not move between the two habitat types (i.e., particle types), whereas DNA is either able to move between the niches (global gene pool version) or not (local gene pools version) in separate versions. Our model belongs to a class of models considering the joint effects of selection, recombination and gene flow (often called migration but referring solely to transfer of DNA here, rather than movement of individuals) between two habitats (Lenormand, 2002; Gavrilets, 2004; Supporting information Appendix S1).

#### 2.1.2 Reproduction and selection

We implement a discrete time Wright‐Fisher model of drift in a haploid population of size *N*, in which each iteration is equivalent to a generation (e.g., as applied to bacteria by Fraser, Hanage, & Spratt, 2007). Simulations follow a single ecological trait determined by *L* loci. A locus can be thought of as a single gene, or a set of tightly linked genes such as an integron or a plasmid (Yeaman and Whitlock 2011). Due to computational limitations, *N *is 2,000 individuals, 1,000 in each subpopulation, and *L *is set to ten loci. The population is encoded as a matrix of size (*N × L*). Each position in the matrix contains the effect size of the allele that individual *i *carries at locus *l*, *a_il_*. Gene effect sizes are on the same arbitrary and continuous scale as the phenotype of each individual, and both can take any value. The phenotype *p *of each individual cell is the sum of the effect sizes *a_il_* at every locus. Thus, gene effects are additive, and the phenotype is fully genetically determined (there is no environmental variation).

Through fission, each individual divides into two identical offspring. From this “offspring pool” of size 2*N*, half survive into the next generation (i.e., *N* remains constant). Survivors are sampled at random; under selection, this sampling is weighted proportional to the fitness of the offspring. Fitness is determined relative to an optimum *x *(in the units of the phenotype), which differs in each niche. A simple model of stabilizing selection was adopted from Lande (1976):

(1) $$W\left(p\right)=\exp-\frac{{\left(p-x\right)}^{2}}{2\sigma_{w}^{2}}$$

where *W*(*p*) is the fitness of phenotype *p*. The fitness of an individual increases as its phenotype approaches the optimum, *x*. The strength of stabilizing selection is determined by $\sigma_{w}^{2}$; the greater its value, the greater the range of phenotypes persists under selection and the weaker selection is.

#### 2.1.3 Mutation

**Figure 1:**
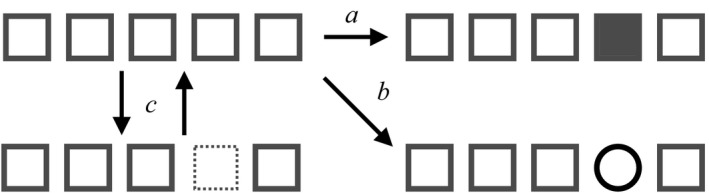
Mutation, recombination, and gene gain or loss as defined in the model, illustrated with five loci. (a) Mutation changes the identity of the allele at a given locus, and the new allele will carry a different effect size (represented by a filled box). (b) Recombination replaces the resident allele with another one from a different individual in the population (represented as a circle). (c) Gene loss results in an empty locus, that can neither recombine nor mutate, but which can be repopulated with the gain of an allele from another individual

Mutation occurs at a rate *m* = 0.005, specified per extant gene copy (Figure 1). We implemented two versions. In the model with fixed effect sizes, each locus has two alleles with value +*a* or −*a, *and symmetrical mutation between them. For example, with 10 loci, all *a_il_* = +0.1 or −0.1. In the model with evolving effect sizes, as in Yeaman and Whitlock (2011), we assumed that mutation changes the effect size of a locus according to a normal distribution centered on the current allele's effect size with variance $\sigma_{m}^{2}$.

#### 2.1.4 Homologous recombination

Homologous recombination occurs at rate *r* and is assumed to be a process of gene conversion, whereby a gene copy (chosen in the same manner as the gene copy to be mutated) is replaced by an allele from the same locus. The replacing allele is chosen at random from a pool containing all extant alleles at that locus (i.e., it is thus possible for a gene copy to recombine with itself). In different versions, we assumed either that gene pools extend globally across the whole population or separately within each subpopulation, that is, from a local, niche‐specific gene pool.

#### 2.1.5 Gene loss and gain

Gene loss (rate *λ*) is implemented in the same way as mutation (Figure 1), but results in the matrix position *a_il_* losing its numerical value: that is, when a locus is lost, its effect size is assumed to be zero. Such loci are exempt from mutation or homologous recombination: we assume that HR requires tracts of homology with incoming DNA, but these are absent after gene loss (Retchless & Lawrence, 2007). In gene gain models, such “empty” locus slots can be re‐occupied by a copy of the equivalent locus from another individual, at a rate *γ*, which is specified per empty locus. If no other individual carries a copy of that particular locus, the locus has gone extinct. Note that we model gene gain as an independent process from HR. In reality, gene gain might occur through HR at flanking regions to the lost gene. The rate of this event would be lower, however, than the rate of HR with the gene present, because there is less homologous sequence present for incoming DNA to match to (Retchless & Lawrence, 2007). Even if the same genetic mechanisms are involved, it is, therefore, appropriate to model gene gain as a different process from HR into a genotype already possessing the gene.

#### 2.1.6 Parameter values

We ran models with three values of recombination rate. *r* = 0.05 is 10× the mutation rate, which is lower than estimates in the *Vibrio* study of Shapiro et al. (2012), but above the level expected to generate quasi‐sexual dynamics in neutral models (Fraser et al. 2007), and within the top 21% of estimates of *r*/*m* across a range of bacteria including other *Vibrio* (Vos & Didelot, 2009). *r* = 0.005 equals the mutation rate, which is at the threshold switching from clonal to quasi‐sexual dynamics in neutral models and close to estimates for bacteria such as *Pseudomonas syringae* and *Enterococcus faecium *(within the top 56% of *r*/*m* in surveyed species, Vos & Didelot, 2009). *r* = 0 is the clonal model, which could not be rejected for 12% of species surveyed by Vos and Didelot (2009). We selected a low rate of gene loss (and gain) at 50× less than mutation rate, *λ* = 0.0001, because pilot simulations showed this was sufficient to demonstrate major effects without slowing down computation time unduly.

Shapiro et al. (2012) did not estimate selection coefficients in the *Vibrio* example, and we are unaware of other bacterial studies of divergence with gene flow that do. Divergence with gene flow is well known to depend on the strength of selection relative to recombination and gene flow (Bürger, 2014; Gavrilets, 2004): we decided to hold selection fixed and vary HR and patterns of gene flow to explore different scenarios. We used a deterministic model to choose parameter values that yielded phenotypic divergence in the clonal model but noticeably reduced phenotypic divergence in a model with recombination and a global gene pool (Supporting information Appendix S1: Figure S1). These conditions allow us to identify which extra features of our simulations are able to promote further divergence. With $\sigma_{w}^{2}$ = 2 in Equation (1), the relative fitness of a genotype with one maladapted allele from the other subpopulation is 0.99 with 10 loci and equal effect sizes (e.g., a phenotype of 0.9–0.1 = 0.8 in the niche with optimum phenotype = 1), falling to 0.78 if the effect is shared equally between just two loci and to 0.37 if the effect is concentrated in one locus. Note that our models only consider symmetrical patterns of selection and gene flow between niches: asymmetry can lead to additional outcomes such as gene swamping (Lenormand, 2002) but our concern here is understanding the impact of gene flow in scenarios where divergence is otherwise able to occur.

### 2.2 Simulations

Each run started with a fully homogenous population with an optimal phenotype for niche 1. After a burn‐in of 1,000 generations of neutral reproduction and mutation (chosen to optimize computation times across all of the simulations), the ancestral population was split into two populations, and the simulation continued for 5,000 generations (flow diagram in Figure S2). Individuals cannot migrate between subpopulations and rates of mutation, homologous recombination, gene gain or gene loss did not differ between niches.

Ten replicate simulations were run for each factorial combination of parameter combinations exploring the effects of different processes: three recombination rates; neutral versus divergent selection; global versus local gene pool; no gene loss, with gene loss, or with both gene loss and gain; and fixed versus evolving effect sizes of loci. Neutral models were not run for models with gene loss as they drift toward the extinction of all loci. Also, because the model starts with a maximum number of loci that can contribute to the trait of interest, the effects of gene gain on its own were not investigated, only in concert with gene loss. Other parameter values are in Table 1. Code development, profiling using profvis (Chang and Luraschi, 2016), and data analysis were performed in R 3.2.3 (R Core Team, 2015), while simulations ran on Imperial College London's high performance computing cluster in R 2.13.0 (R Core Team, 2011).

**Table 1:** Parameter values, their interpretation, and values explored in the cluster simulations

Parameter	Description	Value (s)		
*N*	Population size	2,000		
*l*	Number of loci	10		
*m*	Mutation rate	0.005		
*r*	Recombination rate	0.05	0.005	0.0
*λ*	Rate of gene loss	0.0001	0.0	
*γ*	Rate of gene gain	0.0001	0.0	
$\sigma_{m}^{2}$	Variation in mutation effect size	0.0004		
$\sigma_{w}^{2}$	Width of fitness function (strength of selection)	2.0		
*x*	Environmental optimum	1.0	−1.0	

### 2.3 Differentiation and summary statistics

We measured phenotypic differentiation as the magnitude of (mean phenotype population 1—mean phenotype population 2). Linear models were used to evaluate treatment effects. For simulations in which genome content was variable, the number of extant loci, and the number of extant gene copies per surviving locus were counted at the end of the simulation run.

In models of evolving effect sizes, we followed Yeaman and Whitlock (2011) to capture the degree of divergence among loci. As multiple alleles can exist within a subpopulation, only the most frequent or “leading” allele was considered. Between subpopulations, divergence at a given locus was calculated as the absolute difference in effect size of the leading alleles, *d* = |*a_1_*−*a_2_*| (where *a_1_* and *a_2_* are the leading alleles in each subpopulation). *d* was calculated for every locus, and then further summarized as the divergence of the locus with the largest divergence, *d*_max_, and the mean divergence, *d*_mean_, which were both recorded each generation. The greater the difference between *d*_max_ and *d*_mean_, the more a single locus (namely that of largest effect) contributes to the divergence between both subpopulations. For the simulations involving variation in genome content, gene absences were treated as loci of effect size zero, and can be the “leading allele” when most individuals in a subpopulation have lost that locus.

## 3 RESULTS

### 3.1 Fixed effect sizes and genome content

**Figure 2:**
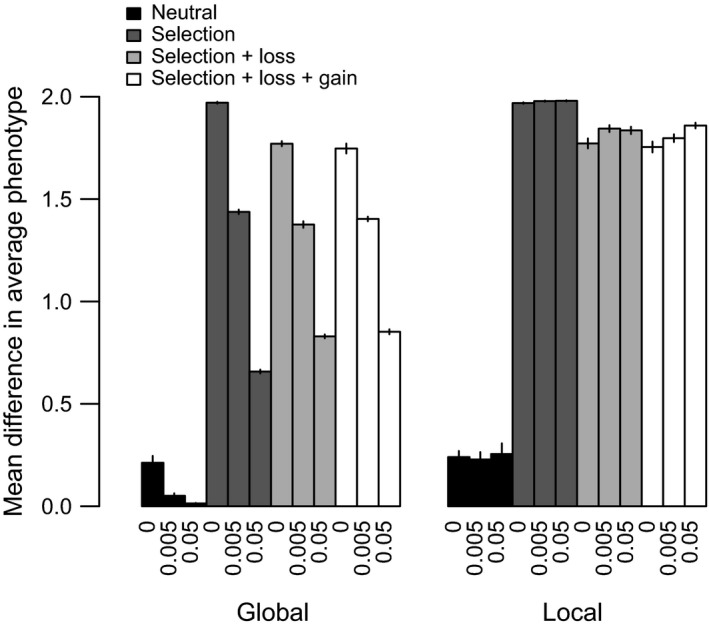
Mean difference in average phenotype between the two populations across simulations with fixed effect sizes of mutations under global versus local gene pool models. Black: neutral; dark gray: divergent selection; light gray: divergent selection plus gene loss; and white: divergent selection plus gene loss and gene gain. Each bar shows the mean and standard error from ten replicate runs. X‐labels show recombination rates. Other parameter values are in Table 1

As a baseline for comparison with models of evolving effect sizes, we first ran a set of models assuming fixed effect sizes (i.e., all *a_il_* = +0.1 or −0.1) and no gene loss or gain. We first report results under neutral conditions (Figure 2, black bars). As expected, in the strict clonal case, the populations diverge due to independent drift caused by lack of migration of cells between them, albeit to a limited extent. With a global gene pool, divergence decreases as recombination rate increases, showing that both recombination rates used here were sufficient to limit neutral divergence under these conditions (linear model of phenotypic divergence vs. recombination rate *r *as factor: *r* = 0.005 vs. *r* = 0, *t* = −5.22, *p* < 0.0001; *r* = 0.05 vs. *r* = 0, *t* = −5.84, *p* < 0.0001, *df* = 57). In contrast, with a local gene pool, there is no longer an effect of recombination rate on divergence—local gene pools by definition entail no gene flow between populations.

With selection to different optima in each subpopulation, phenotypic divergence increases across all parameter combinations compared to neutral simulations (Figure 2, dark gray bars). The strength of selection used here was, therefore, strong enough to promote adaptive divergence across all models: gene flow is never strong enough to reduce divergence to neutral levels. The extent of divergence does vary markedly, however, depending on the other parameters.

**Figure 3:**
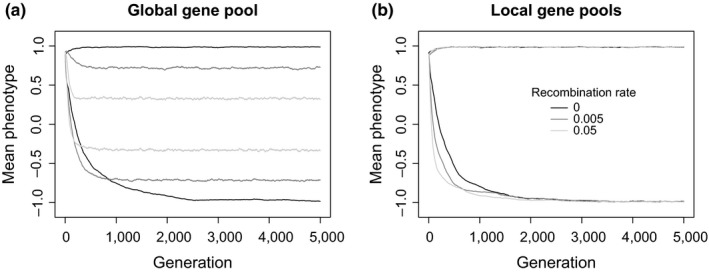
Mean phenotype in population 1 and 2 over time for (a) global versus (b) local gene pools with fixed, equal effect sizes of mutations. In each plot, the top three lines are for population 1 and the bottom three lines are for population 2. Black: clonal; dark gray: recombination rate 0.005; light gray: recombination rate 0.05

Strictly clonal populations diverge the most, falling just short of the expected optimum of two units only because of mutational variation in each population. In the global gene pool model, the degree of divergence falls to almost one third as recombination rate increased. This occurs because gene flow introduces maladaptive alleles from the alternative population, reducing the difference in average phenotypes between the populations. Higher recombination rates within populations lead to faster approaches to the final equilibrium levels of divergence, however, even though the final degree of divergence is reduced due to increased gene flow (Figure 3a). This reflects the widely known benefits of recombination in bringing together beneficial adaptive combinations during adaptation to a new optimum (Burt, 2000; Friedman et al., 2013).

The local gene pool models remove the limiting effects of gene flow on the degree of phenotypic divergence at equilibrium (Figure 2). All models now yield high levels of phenotypic divergence ranging between 1.75 and 2.0 units. There remain some significant effects: simulations with *r* = 0.05 now attain greater phenotypic divergence than the clonal model (*t* = 2.62, *df* = 56, *p* = 0.011). The size of effect is tiny, however (0.017 relative to a mean divergence of 1.97 in the clonal model). The speed of approach to equilibrium remains faster with higher recombination rates (Figure 3b).

These results confirm expectations from earlier models (Gavrilets, 2004; Friedman et al., 2013; and results for a deterministic two‐locus model in the Supporting information Appendix S1: Figure S1) and show that our choice of parameter values provides suitable variation to explore effects of evolving genetic architecture in the next section: the extent of divergence varies both with respect to recombination rate and to global versus local gene pools.

### 3.2 Fixed effects with gene loss and gain

The simulations were repeated with models including either just gene loss or gene loss plus gain in turn, still with fixed and equal effect sizes across loci. Gene gain introduced only minor differences and we, therefore, focus on describing the effects of the model with just gene loss.

**Figure 4:**
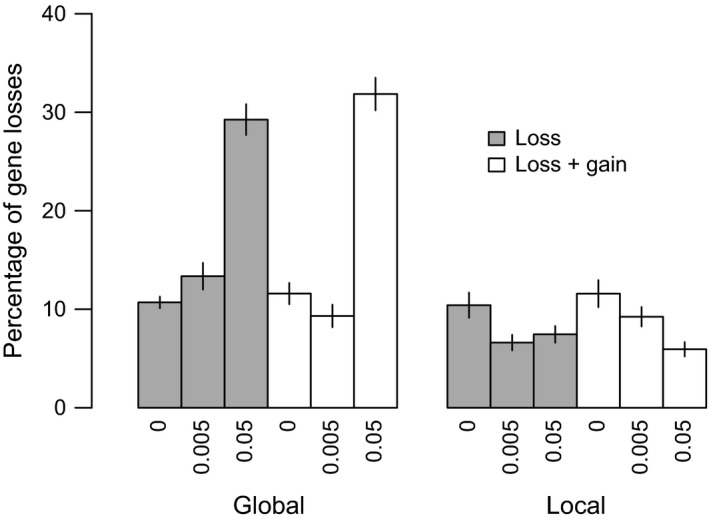
Mean percentage of loci missing across individuals in both populations in the fixed effect size models with divergent selection plus gene loss (gray), and divergent selection plus gene loss and gain (white). X‐labels show recombination rates. Standard errors are shown

With a global gene pool, gene loss reduces the phenotypic divergence in clonal models, reduces phenotypic divergence to a lesser extent when *r* = 0.005, but switches to increase phenotypic divergence when *r* = 0.05, relative to the model with fixed gene content. To unpick these effects, we compared the proportion of loci lost among treatments (Figure 4). A mean of 10% of loci was lost in the clonal model, 13.4% when *r* = 0.005, and 29.2% when *r* = 0.05. Despite selection and the mutational rate of gene loss being the same, the proportion of genes lost, therefore, varies with recombination rate. In the clonal model, an initial loss of a locus may be selectively advantageous in the population with a low optimum, but by the end, it is disadvantageous as the optimal phenotype in each population can only be attained by a full complement of loci. Note that mutation occurs at a faster rate than gene loss, so mutation is expected to dominate in the response to selection.

In contrast, with *r* = 0.05, gene loss can partly reduce the negative effects of gene flow by leading to private genes within each population. The contribution of these loci to the phenotype in each population is protected from the effects of gene flow. On average, 10% of loci are restricted to a single population when *r* = 0 (indicated by a frequency ~0.5), 18% with *r* = 0.005, compared to 53% when *r* = 0.05. With half of the loci lost in one population and the other half lost in the second population, the maximum and minimum attainable phenotypes are 0.5 and −0.5, that is, a divergence of 1.0, but this is closer to the optimum in each niche than is possible with no gene loss and gene flow between populations (Figure 2). Transfer of maladapted genes from the other population, therefore, selects for increased gene loss in the global gene flow model, once recombination is above a threshold rate (which is calculated for a deterministic model with two biallelic loci in the Supporting Information Appendix S1: Figure S3). In turn, gene loss protects local adaptation from the homogenizing effects of gene flow.

With the local gene pool model, the effects of gene loss are reduced and constitute a small decline in phenotypic divergence irrespective of the rate of recombination. In common with the clonal model, the only effect is the loss of some loci during initial divergence: recombination marginally reduces the rate of loss, but the effect is not significant (Figure 4).

### 3.3 Phenotypic divergence with evolving effect sizes

The simulations were next repeated for the model of evolving effect sizes. To allow for the longer timescale to reach stationary conditions, these models ran for 500,000 generations (as in Yeaman and Whitlock 2011). Evolving effect sizes make no difference to phenotypic divergence under neutrality compared to the fixed effect size models. Because the simulation runs were 100 times longer, phenotypic divergence due to drift in the clonal model is on average more than twice that expected under selection by the end (4.21 ± 0.64). Otherwise, results are the same: neutral models with global recombination showed no phenotypic divergence.

**Figure 5:**
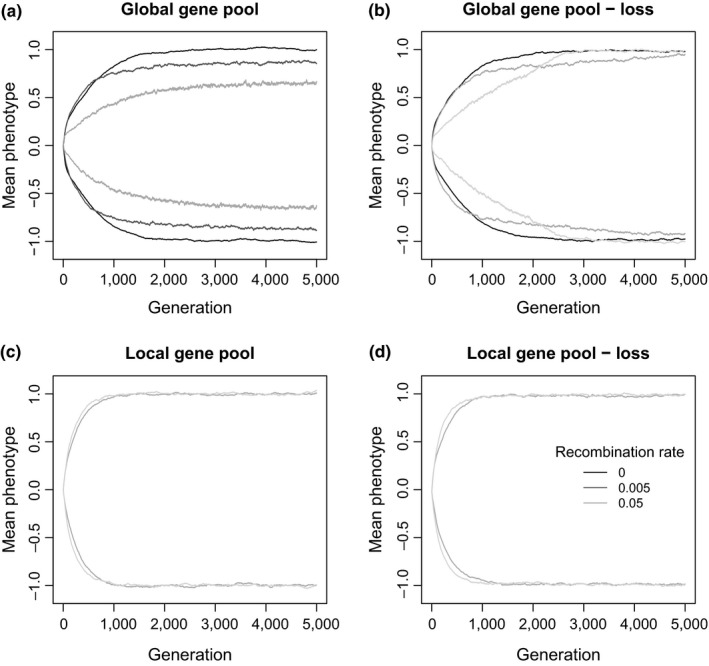
Mean phenotype in simulations under selection with evolving gene effect sizes within the first 5,000 generations for (a) global gene pool, no gene loss, (b) global gene pool with gene loss, (c) local gene pool, no gene loss, and (d) local gene pool with gene loss. In each plot, the top three lines are for population 1 and the bottom three lines are for population 2. Black: clonal; dark gray, recombination rate 0.005; light gray: recombination rate 0.05. Note that, for *r* = 0.005 in plot A, divergence slowly continues beyond the first 5,000 generations to be indistinguishable from *r* = 0

With selection and a global gene pool, the optimal divergence of two phenotypic units is attained in most parameter combinations. Contrary to results with fixed effect sizes, divergence at the end of the simulations no longer decreases in the presence of gene flow or gene loss to the same extent. The one exception is a small but significant decrease with *r* = 0.05 and no gene loss (linear model of phenotypic divergence vs. *r *as factor: *r = *0.005 vs. *r* = 0, *t* = −1.216, *p* > 0.05; *r = *0.05 vs. *r = *0, *t* = −9.473, *p* < 0.005, *df* = 47, Figure 5a). This effect disappears in the presence of gene loss. The evolution of gene effect sizes, therefore, enhances phenotypic adaptation by reducing both the homogenizing effects of gene flow and the mutational load from gene loss compared to models with fixed effect sizes. Consequently, whether gene flow is local or global no longer makes a difference to the final degree of phenotypic divergence.

Evolving effect sizes also interact with gene loss to change the shorter‐term dynamics of phenotypic divergence. With gene loss and high recombination rate, population divergence is initially slow, but divergence continues until the optimum divergence is reached (Figure 5b, light gray line). In contrast, populations with an intermediate recombination rate diverge faster initially, but then reach the optimum far later (Figure 5b, dark gray line).

### 3.4 Genetic divergence with evolving effect sizes: fixed genome content

**Figure 6:**
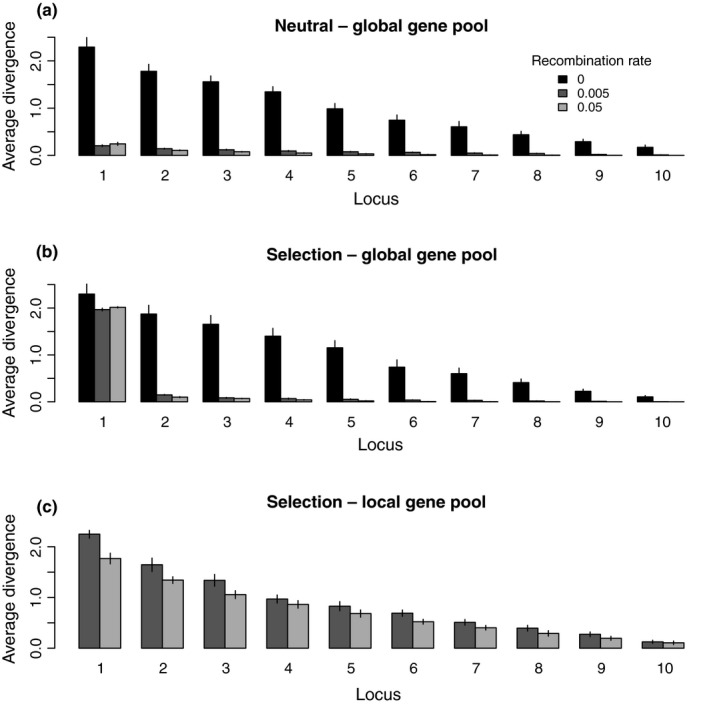
Average divergence in gene effects per locus in simulations with freely evolving effect sizes but no loss of genes. (a) Neutral model. (b) Selection with global gene pool. (c) Selection with local gene pool Loci are sorted according to effect size from gene with largest effect (left) to the gene with the smallest effect (right)

To further understand these patterns, we looked at how gene effect sizes changed across loci and among treatments, first under neutral conditions. Gene effects diverge at every locus in the clonal model, declining gradually from the locus of largest divergence to the lowest (Figure 6a, black bars). As expected, divergence remains low across all loci in the presence of recombination and global gene flow (Figure 6a, gray bars). Divergence decreases with increasing recombination rate (and hence gene flow) across loci (multivariate linear model of *d* at each locus, sorted by divergence, against recombination rate in neutral simulations with recombination: *F*_1,18_ = 2.75, *p* > 0.05).

With selection and global gene flow, the divergence *d* observed at each locus depends strongly on the recombination rate. In strictly clonal populations (Figure 6b, black bars), the distribution of divergences across loci is equivalent to that under neutrality (multivariate linear model of *d* at each locus, sorted by divergence, against presence or absence of selection in simulations with no recombination: *F*_1,18_ = 0.453, *p* > 0.1), even though the resulting phenotypes are very different. This is a consequence of our assumption of additive gene effects; the phenotype is the sum of the effects of each locus. As a locus can have positive as well as negative effects, the only constraint selection imposes is that the gene effects sum to a value close to the optimum. With a high mutation rate and many loci, a mildly deleterious mutation in one locus can be easily offset by a mutation elsewhere, resulting in a nearly neutral accumulation of mutations (see also Yeaman & Whitlock, 2011). Consequently, the locus of largest divergence increases steadily in effect throughout the simulation (Supporting information Figure S4, black line), and is compensated by other loci when its divergence exceeds the optimum value.

With recombination and a global gene pool, divergence is concentrated in a single locus (Figure 6b, gray bars, locus 1), and divergence between populations is suppressed in other loci. The shift toward a single major locus minimizes the transfer of maladaptive alleles and thereby optimizes adaptation to each environmental optimum. Divergence at the other, minor loci is more strongly suppressed at higher rates of recombination (and hence gene flow). A deterministic model of two biallelic loci confirms that with a global gene pool and no gene loss, the greatest attainable divergence (and hence mean fitness) is with two locally adapted alleles at a single locus (i.e., alleles A and a at the major locus have effect sizes +1.0 and −1.0, alleles at other loci have effect size of 0, Supporting information Figure S5). This is because selection more efficiently removes maladapted genes arriving into each population: no intermediate phenotypes arise that are less strongly selected against but carry maladapted alleles (Supporting information Appendix S1). The benefit increases at higher *r* (Supporting information Figure S5), hence the stronger concentration into a single locus when *r* = 0.05 than when *r* = 0.005 in the simulations. But phenotypic divergence still decreases at high *r*, explaining why the scenario of evolving effect with *r* = 0.05 and no gene loss had phenotypic divergence significantly below the optimum of two.

This distribution of gene effect sizes on divergence is reached quickly at the high rate of recombination and more slowly when the recombination rate is intermediate (Supporting information Figure S4a, light gray and dark gray lines, respectively), because of stronger selection for concentrating effects into a single locus at higher *r*. In fact, when *r* = 0.005, the locus of largest divergence (*d*_max_) spends varying amounts of time at an intermediate level of divergence about half‐way to the equilibrium point (Supporting information Figure S6). On average, it took each replicate simulation roughly 200,000 ± 40,000 generations to reach the equilibrium level, with a range of roughly 40,000–400,000 generations. This occurs because the populations become “stuck” at a local optimum of two loci, each with two alleles locally adapted to each niche, that is, locus A with allele effect sizes +0.5 and −0.5, and locus B with allele effect sizes +0.5 and −0.5. Any initial single mutation to progress from that genotype to the optimal genotype (i.e., a single A locus with allele effect sizes +1 and −1) leads to a loss of mean fitness: only a few combinations of 2 or 4 mutations lead to increased mean fitness (Supporting information Figure S7). In contrast, populations do not become stuck at the local optimum when *r* = 0.05 because (a) there is weaker selection against single mutants, (b) high recombination leads to faster production of beneficial combinations and (c) high recombination rate leads to stronger selection pressure for concentrating gene effects into a single locus (Supporting information Figure S7).

In contrast, recombination and local gene pools (Figure 6c) lead to a diffusion of divergence throughout the genome similar to that observed in strictly clonal populations. The differences between the local and global gene pool models mirror predictions in sexual eukaryotes: allopatric speciation results in even divergence across the genome whereas speciation with gene flow leads to divergence concentrated in genomic islands under selection (Ellegren et al. 2012).

Note that, although there is no gene flow between niches in the local gene pool model, divergence across the genome is lower at the higher recombination rate. Runs with *r* = 0.005 display divergence patterns very similar to the clonal model: the locus with greatest divergence steadily increases in divergence over time with compensation at other loci (Supporting information Figure S4b, dark gray line). In contrast, with *r* = 0.05, divergence at the locus with the greatest divergence slows down over time. Once the optimum phenotypic divergence is reached, *r* = 0.05 selects against new mutations with large effects and subsequent compensating mutations because these are incompatible with the prevailing genetic background, which they are tested against more frequently than when *r* = 0.005. This demonstrates additional effects of HR within populations on the evolution of genetic architecture.

### 3.5 Genetic divergence with evolving effect sizes: gene loss and gain

With gene loss and gene gain, by the end of the simulation on average, 88.6% of genes are lost from both populations, irrespective of treatment. As above, these results are to be expected under our model of additive gene effects and high mutation rate, whereby one locus can evolve to replace the phenotypic effect of all other loci. As before, gene gain had little effect.

Upon closer inspection, 71% of simulation runs involving loss ended with only two loci remaining, and only one run ended with as many as four surviving loci. Each surviving locus was found on average in 1,019 cells. This implies that for the majority of simulations, only a single locus survives in each subpopulation (of size 1,000 individuals), and that these are usually different loci. Among the simulations involving local gene pools, however, there were three runs that had only a single locus remaining (i.e., the same locus in both populations).

Again, these results can be interpreted using a simpler model of two biallelic loci (Supporting information Appendix S1: Figure S5). Comparing scenarios with global gene flow and with and without gene loss, the optimal divergence is obtained when effects are partitioned between loci, that is, cells in population 1 have a locus with alleles a and A with effect sizes +1 and 0, whereas population 2 has a different locus with alleles b and B with effect sizes −1 and 0. No transfer of maladapted alleles can occur because HR cannot occur between different loci. At low recombination rates, the benefit over the single locus, specialist allele solution that evolves in the absence of gene loss is minimal, but it becomes greater at higher recombination rates: hence the lower phenotypic divergence without gene loss than with gene loss when *r* = 0.05.

**Figure 7:**
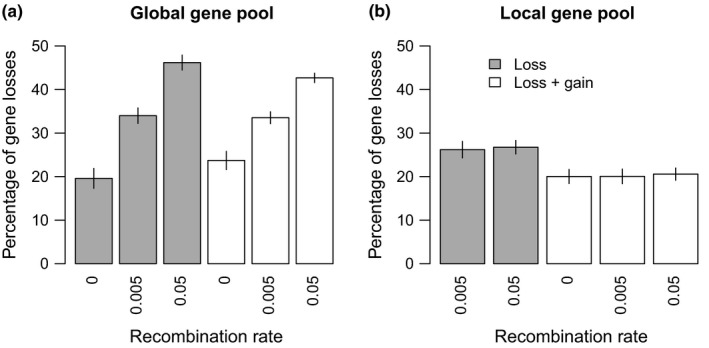
Percentage of loci missing within 5,000 generations across all individuals in the models with evolving effect sizes. (a) Global gene pool. (b) Local gene pool

If we consider the dynamics of gene loss earlier in the simulation (Figure 7 and Supporting information Figure S8), treatment has a clear effect on the rate of gene loss. Differences in the percentage of genes lost are visible within 5,000 generations, with higher recombination rates increasing the rate of loss of genes in simulations with a global gene pool (Figure 7a), irrespective of gene gain. This effect is notably absent from simulations with a local gene pool (Figure 7b). However, gene gain slows down the loss of genes in the latter case (where gene gain draws from a local gene pool of locally adapted alleles), an effect that is also visible but not as pronounced when gene gain is global.

## 4 DISCUSSION

We explored the role of genetic architecture in the ecological divergence of bacterial populations across a spectrum of recombination rates (zero, equal to mutation rate and greater than mutation rate) that are broadly representative of those observed in nature. In the models with recombination, we also considered divergence with local versus global gene pools.

Our models show that evolving genetic architecture can aid divergence in bacteria in the face of recombination and gene flow. The transfer of maladapted genes into locally adapted genetic backgrounds generated selection for gene effects to be concentrated into as few loci as possible. The same concentration of effects was not observed with niche‐specific gene pools, as then there is no influx of locally maladapted genes from the other population. Genetic architecture versus niche‐specific gene pools is therefore alternative mechanisms for limiting the constraining effects of gene flow. Niche‐specific gene pools allow populations to differentiate more rapidly than a scenario with evolving genetic architecture (which took 100,000s of generations with the intermediate recombination rate). But evolving genetic architecture broadens the range of environmental conditions under which divergence can occur to include those without niche‐specific restrictions to gene flow. Niche‐specific gene pools are not required but definitely helpful for ecological divergence.

These conclusions extend the results of Friedman et al. (2013), who predicted a decreasing probability of divergence with gene flow in bacteria with increasing number of loci. They also mirror the findings of models of divergence with gene flow in sexual organisms (Gavrilets, 2004; Yeaman & Whitlock, 2011). HR is not completely equivalent to meiotic recombination, because it exposes a single maladapted gene region arriving by gene flow to selection in an otherwise fully adapted genome. In clonal and niche‐specific gene pool models, we observed the evolution of co‐adapted sets of loci with complementary gene effects, and there is scope for further exploration of how this might vary with meiotic shuffling versus HR. But results for effects of gene flow on divergence are qualitatively similar to in sexual organisms.

Testing these qualitative predictions will require more systematic studies of bacterial divergence in nature and in the lab. Unfortunately, there has been surprisingly little research into the genetic architecture of bacterial traits. Although there is some evidence for the sort of additive gene effects we modeled (Abe & Benedetti, 2016; Hunter & Keener, 2014), many traits display more complex interactions (Brbić et al., 2016). However, we believe our qualitative predictions are likely to hold at the sequence level as well. For example, in the motivating study for our model, Shapiro et al. (2012) characterized the genomes of two recently diverged populations of the marine bacterium *Vibrio cyclitrophicus*. They found evidence of high homologous recombination (enough to obscure any signal of clonality), and a strong trend for recent recombination to happen mainly within, rather than between populations. The single nucleotide polymorphisms (SNPs) that supported ecological differentiation between the two populations were spread among 11 loci, of which three accounted for over 80% of SNPs. Although this distribution is consistent with the qualitative predictions of both our model and that of Friedman et al. (2013), it is also possible they reflect other mechanisms influencing the evolvability of these loci. Selection experiments of divergence in laboratory bacteria, with and without gene flow, would also be a useful approach for testing the evolution of different processes described here. Although we have emphasized recombination and gene flow here, evaluation should consider the range of recombination rates observed across bacteria—many of which are indeed effectively clonal and ecotype models of divergence should apply.

Our models also show that gene loss can further protect locally adapted genes from an influx of maladapted genes by leading to private loci adapted to each environment. Gene loss is a well‐known phenomenon in bacteria and is often interpreted in terms of specialization: generalists carry many genes to enable metabolic flexibility, but specialists lose costly genes that are unnecessary in their specific environment (Koskiniemi, Sun, Berg, & Andersson, 2012). Especially in bacteria with little or no horizontal gene transfer, phenotypic diversity between strains can originate through differential loss of genes, as found in the highly clonal *Mycobacterium tuberculosis* species complex (Bolotin & Hershberg, 2015). Our model highlights a new potential force on gene loss in bacteria, namely that differential gene loss could be intensified when two differentiating specialists maintain the potential to exchange DNA.

The comparison of patterns of divergence between *E. coli* and *Salmonella enterica* across genome regions by Retchless and Lawrence (2007) provides a possible example of this mechanism. Divergence varied considerably among genome regions, with some parts diverging over 168 Mya whereas others diverged up to 70 My later. The earliest diverging regions included genes that produced structures on the surface of the cell known to be under frequency‐dependent or diversifying selection—that is, niche‐specific genes—but also regions exhibiting differences in gene content. The loss of different genes in each lineage was hypothesized to limit the potential for recombination and gene flow in surrounding regions of the genome during the early stages of ecological divergence, whereas gene flow could continue between contiguous regions of homology in the background genome. Similarly, ecologically diverging populations of a thermophilic cyanobacterium (*Mastigocladus laminosus*) display low differentiation across most of the genome—indicative of ongoing gene flow via HR. Divergence is concentrated in a genome region associated with production of the protective envelope of the heterocyst, and is associated with the deletion of two genes. While gene loss might be favoured through direct effects on the heterocyst phenotype, reduction of gene flow in this genome region in order to maintain ecologically adapted genotypes could also play a role, as predicted by our model.

Our gene‐loss mechanism requires that multiple, exchangeable loci contribute to variation in a trait under differential selection between populations. For example, changes in the utilization of a particular carbon source could arise through multiple changes in underlying enzymes involved in its metabolism and the regulatory pathways that control them. If divergent selection acted instead on a set of multiple traits each coded by separate loci that are all essential for cellular function, then clearly gene loss could not aid divergence. A more realistic version of our model could incorporate loci targeted by different components of selection that are either shared or divergent between populations. It remains to be seen if the predicted interaction between high rates of HR plus gene flow and gene loss will hold as more empirical studies on the genomics of bacterial divergence in sympatry become available. Cases where the selective advantage of having a given locus is outweighed by the disadvantage of high gene flow at that locus may turn out to be rare in nature. However, in the lab, bacteria can quickly evolve compensatory mutations and restore growth, even after the loss of a core glycolysis gene (Charusanti et al., 2010).

In contrast to gene loss, gene gain had little impact in our simulations. This is consistent with the idea that gene gain is only rarely selectively advantageous and largely plays a role in adaptation to a novel niche. For example, Niehus et al. (2015) showed that horizontal transmission of genes dominates a population (or community) only insofar as there is an influx of nonadapted strains lacking an ecologically relevant gene. In the absence of migration, the selective advantage of individuals with the gene in question is large enough for transmission to be almost exclusively driven vertically. The limited effects of gene gain also demonstrate that the emergence of private genes in our gene‐loss models was not an artifact of our assumption that genes cannot re‐enter the population once lost.

Further mechanisms not considered here could be incorporated into future models. In interpreting our model, each “locus” could be either one or multiple genes. Thus, the concentration of gene effects we report can be seen either as a consequence of cumulative mutations in one gene, or the concentration of multiple previously dispersed genes into, for example, a single operon or integron (see also Yeaman & Whitlock, 2011). A future modeling effort may wish to distinguish between these two processes; we postulate that a process involving genome rearrangements would result in similar concentration of gene effects over time.

A final mechanism for reducing maladaptive gene flow between ecologically divergent populations implied by our model is the reduction in recombination rates. Under the static conditions modelled here, there was a transient benefit to recombination within populations in promoting divergence, but once populations approached the new optimum, clonal populations always attained the maximum optimum divergence. If recombination rate itself were under selection, it might decline in order to maintain divergence under static conditions. Different kinds of fluctuating conditions and combinations of shared versus local optima between populations might shift the balance among different mechanisms for reducing gene flow.

## 5 CONCLUSION

Ecological divergence in prokaryotes involves variation in both allele and genome content. We integrated both components into a population model of bacteria to understand their influence on divergence on a gene by gene basis. Both gene flow through homologous recombination and gene loss favored the concentration of divergence into as few loci as possible, thus limiting gene flow between diverging populations. In the absence of recombination and gene flow, genes evolve co‐dependency, and divergence is carried by multiple loci. These predictions need to be tested empirically across bacteria with a wide range of recombination rates.

## AUTHOR CONTRIBUTIONS

Both authors devised the study, programed the model, ran simulations, performed analyses and wrote up the manuscript.

## Data Availability

All scripts for generating and analyzing simulation outputs: Dryad doi: https://doi.org/10.5061/dryad.d53g10q.
