# Supplementary material for: The role of recombination, niche‐specific gene pools and flexible genomes in the ecological speciation of bacteria
Source: Ecol Evol. 2019 Apr 4;9(8):4544–56. doi: 10.1002/ece3.5052 (PMC6476844; doi:10.1002/ece3.5052)
Supplement: Supplementary file 1 [file ECE3-9-4544-s001.docx]

### Supporting information

### APPENDIX

We here present an analytical model to demonstrate the general effects of parameters on divergence under different scenarios explored in the simulation models.

*1. Analytical results for single locus model*

We first analyzed results for a one-locus deterministic model in discrete time. There are two alleles, A and a, where A yields phenotype 1, which is optimal in sub-population 1, and allele a yields phenotype -1, which is optimum in sub-population 2. The relative fitnesses and frequencies of each allele in each sub-population are:

| Genotype | Phenotype | Fitness  Subpop 1 | Fitness  Subpop 2 | Frequency  Subpop 1 | Frequency  Subpop 2 |
| --- | --- | --- | --- | --- | --- |
| A | 1 | 1 | 1 - *s* | *p_1_* | *p_2_* |
| a | -1 | 1 - *s* | 1 | 1- *p_1_* | 1- *p_2_* |

where *s* is the selection coefficient. The change in frequency of A in sub-population 1 due mutation occurring at rate *m* is

*Δp_1_* = *m*(1- *p_1_*)-*mp_1_* = *m(*1*-2p_1_*)

The change in frequency due to gene conversion at rate *r* depends on the type of model. With a local, niche-specific gene pool, gene conversion only occurs separately within each population, i.e.

*Δp_1_* = *-rp_1_*(1 - *p_1_*) *+ r(1-p_1_*)*p_1_* = *0*

which is the same as the strictly clonal model with *r*=0, i.e. it has no net effect. With a global gene pool, cells with A are pasted over by allele a at a frequency determined by the average frequency of a in both sub-populations, i.e. ((1- *p_1_*)+ (1- *p_2_*))/2, yielding

*Δp_1_* = *-0.5rp_1_*(1 - *p_1_* + 1 – *p_2_*) *+ 0.5r(1-p_1_*)(*p_1_* + p*_2_*)

= 0.5*r* (*p_1_* – *p_2_*)

Finally, selection

*Δp_1_* = *p_1_*/(*p_1_* + (1-*s*)(1 – *p_1_*)) *- p_1_*

= *sp_1_*(1- *p_1_*)/(*p_1_* + (1-*s*)(1 – *p_1_*))

$\approx$ *s*(1- *p_1_*)

The overall change in frequency of A in sub-population 1 per generation is therefore

*Δp_1_* = *m(*1*-2p_1_*) + *s*(1- *p_1_*)

for the local gene-pool model and

*Δp_1_* = *m(*1*-2p_1_*) + 0.5*r* (*p_1_* – *p_2_*) + *s*(1- *p_1_*)

for the global gene-pool. These can be solved at equilibrium using the additional observation that *p_1_* = 1- *p_2_* because of the symmetry of the model: allele A reaches the same frequency in sub-population 1 as allele a does in sub-population 2. At equilibrium therefore

$$p_{1}^{*}=\frac{(m+s)}{(2m+s)}$$

for the local gene pool model (equivalent to the clonal model with no gene conversion) and the equilibrium phenotypic divergence between the two sub-populations is

$$D=\frac{2s}{2m+s}$$

whereas for the global gene pool model

$$p_{1}^{*}=\frac{(2m+r+2s)}{2(2m+r+s)}$$

$$D= 4p_{1}^{*}-2 =\frac{2s}{2m+r+s}$$

Gene flow between the sub-populations via a shared gene pool and homologous recombination therefore reduces divergence in an analogous way to migrational load in eukaryotes (Lenormand 2002). The effect is strongest when selection is weaker (fig. S1). There is no recombination *per se* in a single locus model but gene conversion allows ‘migrated’ DNA to enter the population, which permits transfer of locally maladapted alleles between populations.

*2. Analytical results for a deterministic two-locus model*

We now assume the same phenotypic optima in each sub-population, but the trait is determined by two loci with alleles A and a, and B and b respectively, which are initially assumed to be additive and have equal effects. The relative fitnesses are such that the fitness of an optimum genotype in niche *i* finding itself in sub-population *j* is 1-*s*, irrespective of the number of loci underlying the trait; hence number of loci reflects the genetic architecture of the trait.

Genotypes AB, Ab, aB and ab therefore have phenotypes, fitnesses and frequencies of:

| Genotype | Phenotype | Fitness subpop 1 | Fitness subpop 2 | Frequency subpop 1 | Frequency subpop 2 |
| --- | --- | --- | --- | --- | --- |
| AB | 1 | 1 | 1 | *x_1_* | *y_1_* |
| Ab | 0 | 1 – *hs* | 1 – *hs* | *x_2_* | *y_2_* |
| aB | 0 | 1 – *hs* | 1 – *hs* | *x_3_* | *y_3_* |
| ab | -1 | 1 - *s* | 1 - *s* | *x_4_* | *y_4_* |

Gene conversion occurs at a per-locus rate *r*, with frequencies of each type of change calculated as the product of the frequency of each recipient genotype multiplied by the frequency of each allele in the gene pool. For example, the frequency of genotype AB is *x_1_*, the frequency of allele a is *x_3_* + *x_4_* in the local gene pool model, so the rate of conversion of AB to aB is *rx_1_*(*x_3_* + *x_4_*) and the overall change in *x_1_* due to gene conversion is:

*Δx_1_* = *rx_2_*(*x_1_* + *x_3_*) + *rx_3_*(*x_1_* + *x_2_*) - *rx_1_*(*x_2_* + *x_4_*) - *rx_1_*(*x_3_* + *x_4_*)

= 2*r*(*x_2_x_3_* – *x_1_x_4_*)

Similarly, in the global gene pool model, the frequencies of each allele in the global gene pool are the average of the frequencies in the two sub-populations, hence

*Δx_1_* = 0.5*rx_2_*(*x_1_* + *x_3_* + *y_1_* + *y_3_*) + 0.5*rx_3_*(*x_1_* + *x_2_* + *y_1_* + *y_2_*) – 0.5*rx_1_*(*x_2_* + *x_4_* + *y_2_* + *y_4_*) –

0.5*rx_1_*(*x_3_* + *x_4_* + *y_3_* + *y_4_*)

= 0.5*rx_2_*(*2x_3_* + *y_1_* + *y_3_*) + 0.5*rx_3_*(*y_1_* + *y_2_*) - 0.5*rx_1_*(2*x_4_* + *y_2_* + *y_3_* + 2*y_4_*)

Recombination therefore now has a different effect from just gene flow alone (i.e. without recombination, *r*=0) because recombinant genotypes are produced between the two loci.

The change in frequency due to selection is:

*Δx_1_* = *sx_1_*((*1-h*)*x_4_ - hx_1_+h*) */*(*1 - s*((*1-h)x_4_ -hx_1_+h*))

*Δx_2_* = *sx_2_*((*1-h*)*x_4_ - hx_1_*) */*(*1 - s* ((*1-h*)*x_4_ -hx_1_+h*))

*Δx_3_* = *sx_3_*((*1-h*)*x_4_ - hx_1_*) */*(*1 - s* ((*1-h*)*x_4_ -hx_1_+h*))

*Δx_4_* = *sx_4_*((*1-h*)*x_4_ - hx_1_+h*-1) */*(*1 - s*((*1-h)x_4_ -hx_1_+h*))

The following linearization yields equations that can be solved and give close correspondence to simulations of the exact model:

*Δx_1_* $\approx$ *hs*(*1-x_1_*), *Δx_2_* $\approx$ -*hsx_2_*, *Δx_3_* $\approx$ -*hsx_3_*, *Δx_4_* $\approx$ -*hsx_4_*

With this approximation and plugging together with mutation and gene conversion components, the phenotypic divergence in the local gene pool model is

$$D=\frac{2hs}{2m+hs}$$

and in the global gene pool model

$$D=\frac{2hs}{2m+hs+r}$$

Exact simulation results are plotted next to those for the single-locus model in figure S1. Distributing the phenotype between two loci with equal effect but the same combined effects on selection as the single-locus case results in even greater reduction of phenotypic divergence because of gene flow plus recombination than in the single locus model. Simulations results for multilocus versions of the model are in the Results and figure 2.

*3. Gene loss model with two loci and equal effect sizes*

To compare the mean fitness and phenotypic divergence in a scenario with gene loss and private genes, we consider a situation in which genotypes A0 and a0 are segregating in population 1 and 0b and 0B are segregating in population 2 with frequencies and fitness as follows:

| Genotype | Phenotype | Fitness subpop 1 | Fitness subpop 2 | Frequency subpop 1 | Frequency subpop 2 |
| --- | --- | --- | --- | --- | --- |
| A0 | 0.5 | 1 - *j* |  | *p_1_* |  |
| a0 | -0.5 | 1 – *j-k* |  | *1 - p_1_* |  |
| 0B | 0.5 |  | 1 – *j-k* |  | *p_2_* |
| 0b | -0.5 |  | 1 - *j* |  | *1- p_2_* |

The equilibrium frequency of A0 in sub-population 1 reduces to a single locus, bi-allelic solution outlined above, i.e:

$$p_{1}^{*}=\frac{(m+k)}{(2m+k)}$$

with the symmetrical solution for sub-population 2, and phenotypic divergence between the populations, *D*, given by

$$\frac{k}{(2m+k)}$$

Phenotypic divergence, and mean fitness, is higher than the two-locus model with global gene flow and recombination between populations when

$$r>\frac{s(hk-2km-4hm)}{k}$$

There is always a positive effect of gene loss on divergence but it is small/negligible for *r*=0.005 and substantial when *r*=0.005 (appendix figure S3): consistent with the switching effect of gene loss on divergence when *r*=0.05 than when *r*=0.005 in the simulations with 10 loci (although the magnitudes of effect differ from the 2-locus case).

*4. Evolving effect sizes*

The degree of phenotypic divergence attainable under different simple scenarios of altered effect sizes can be used to evaluate simulation results with evolving effect sizes under global gene flow.

*Scenario 1*: *Equal allele effect sizes* of A=+0.5, a=-0.5, B=+0.5, b=-0.5 and no gene loss lead to the divergence calculated above of 2*hs*/(2*m*+*hs*+*r*) (appendix section 2). With loss of locus B in sub-population1 and locus A in sub-population 2, divergence is *k*/(2*m*+*k*), where *k* is the difference in fitness between A0 and a0 in sub-population 1 (appendix section 3). This can either increase or decreases divergence relative to the equivalent scenario with no gene loss (fig. S3 and S5).

*Scenario 2*: *Effects partitioned symmetrically between loci*, i.e. A=1, a=0, B=0 and b=-1, and no gene loss scenario yields the same phenotypes as scenario 1, i.e. phenotypes AB=+1, aB and Ab = 0, ab= -1, and hence the same degree of divergence between the two sub-populations. Partitioning phenotypic effects into different loci has no effect on divergence with gene flow. With loss of locus B in sub-population 1 and locus A in sub-population 2, now the difference in fitness between A0 and a0 is *s* (i.e. phenotypes +1 and -1 in an environment with optimum +1) and therefore divergence is 2*s*/(2*m*+*s*), i.e. equivalent to a local single locus model (appendix section 1). This divergence is always greater than the divergence with no gene loss for the same effect sizes, or indeed the divergence under any other scenario considered for two loci with or without gene loss (fig. S5).

*Scenario 3*: *Effects partitioned between alleles at one locus*, i.e. A=1, a=-1, B=0, b=0, and no gene loss reduces to a single locus model with gene flow and yields a divergence *D*=2*s*/(2*m*+*r*+*s*) that is always greater than the divergence of ‘no gene loss’ in scenarios 1 or 2 (when *h*<1). Concentrating phenotypic effects into a single locus therefore yields greater divergence and higher mean fitness than partitioning effects between loci when there is no gene loss. This is because selection more efficiently removes maladapted genes arriving into each population: individuals are either optimal to niche 1 or to niche 2 but with no intermediate phenotypes that carry maladapted genes but are less strongly selected against (by a fraction *h*). Symmetrical gene loss is in this scenario limits sub-population 2 to a phenotype of 0, yielding a phenotypic divergence between populations of *s*/(2*m*+*s*). This is only favored when *r* > 2*m*+*s*, i.e. when divergence without gene loss is strongly impaired by inflow of maladapted alleles.

|  | Scenario | Effect sizes | | | | Equilibrium divergence, *D* | |
| --- | --- | --- | --- | --- | --- | --- | --- |
|  |  | A | a | B | b | No gene loss | Gene loss |
| 1 | Equal allele effect sizes | 0.5 | -0.5 | 0.5 | -0.5 | 2*hs*/(2*m*+*hs*+*r*) | *k*/(2*m*+*k*) |
| 2 | Effects partitioned between loci | 1.0 | 0 | 0 | -1.0 | 2*hs*/(2*m*+*hs*+*r*) | 2*s*/(2*m*+*s*) |
| 3 | Effects partitioned between alleles at one locus | 1.0 | -1.0 | 0 | 0 | 2*s*/(2*m*+*r*+*s*) | *s*/(2*m*+*s*) |

**Supplementary figure legends**

**Figure S1**. Phenotypic divergence in single (first column) and two-locus (second column) deterministic models with local (first row) versus global (second row) gene pool models. Each line shows simulation of the exact model (rather than approximate solution) for a different selection coefficient, *s*. *h*=0.35 in line with parameter values for the Gaussian function relating phenotype to fitness used in the paper. Full details of models provided in the appendix.

**Figure S2.** Schematic representation of the simulations run with evolving effect sizes on the high performance computing cluster. Each simulation starts with identical clones and goes through 1000 generations of mutation-drift to provide some initial variability. The next phase carries on for 500,000 generations, Mutation and – if present – recombination, gene gain and gene loss are modelled population wide, reproduction and selection is limited to each subpopulation. The average phenotype is recorded for each subpopulation, all other statistics across the entire population.

**Figure S3**. The difference between phenotypic divergence in a two-locus deterministic model with global gene flow and gene loss and phenotypic divergence in the equivalent model without gene loss, in both cases with allele effect sizes A=0.5, B=0.5, a=-0.5, b=-0.5. Recombination rate shown on a log scale, r=0.005 and r=0.05 were values used in 10 locus simulations in main text. Curves from red to indigo in rainbow order indicate results for *s*=0.01, 0.02, 0.05, 0.1, 0.2, 0.4, respectively. Model details provided in the Appendix.

**Figure S4.** Mean divergence at the locus of largest effect (*d_max_*) in simulation models with freely evolving effect sizes, averaged across replicates each generation. Only models including selection are shown. To improve visibility not all plots are to the same scale. Model details provided in the Methods and Results.

**Figure S5**. Comparison of phenotypic divergence under three scenarios in the deterministic model – equal effect sizes, specialist loci and specialist alleles, corresponding to scenarios 1 to 3 in the appendix text – each with and without gene loss. Results for 3 representative selection pressures against the optimal genotypes for subpopulation 1 in subpopulation 2 (and vice versa) are shown. Note that the equal effects and specialist loci scenarios are equivalent with no gene loss. Model details provided in the Appendix.

**Figure S6.** Example trajectory of the divergence at the locus of largest divergence (*d_max_*) in a simulation with *r* = *m*, evolving effect sizes and no gene loss. In this example, maximum divergence remains at an intermediate level for about 200,000 generations before rising to the equilibrium point, at which the locus carries all divergence in phenotype. This behavior is observed only under these conditions.

**Figure S7**. The effects of mutation on mean fitness at equilibrium of a two-locus model simulated with global gene pool and equal symmetrical effect sizes (A=0.5, a=-0.5, B=0.5, b=-0.5), which corresponds to the temporary local optimum observed in simulations of the full model when r=0.005 (fig. S6). The black point shows mean fitness for simulation with the above allele effect sizes: circle for r=0.005, square for r=0.05. Other points show results for all combinations of +0.1 and -0.1 mutations to those allele effect sizes: red = mutation to just one allele (e.g. A=0.6, a=-0.5, B=0.5, b=-0.5); green = double mutants (e.g. A=0.6, a=-0.6, B=0.5, b=-0.5); blue = triple mutants; cyan = quadruple mutants. Mean fitness is always lower when r=0.05 than r=0.005, because of uptake of maladaptive genes into the populations. Single mutants in red always result in lower mean fitness than the local optimum (black): but the reduction in mean fitness is less on average when r=0.05 (average loss in mean fitness = 0.0010) than when r=0.005 (average loss in mean fitness = 0.0012). Only 2 and 4 mutant models result in higher mean fitness (shaded points), and the mean improvement of those is greater when r=0.05 (average change in mean fitness = 0.0006) than when r=0.005 (average change in mean fitness = 0.00006). Together with the well-known effects of higher r for promoting the spread of beneficial gene combinations and lower mean fitness of the local optimum when r=0.05, this explains why the local optimum persists for long timescales in the r=0.005 simulations but not when r=0.05.

**Figure S8**. Dynamics of gene loss within the first 50,000 generations for the models with evolving effect sizes. The black line (*r* = 0) has been repeated in panel A and B for comparison.

**FIGURE S1**

**FIGURE S2**

FIGURE S3

**FIGURE S4**


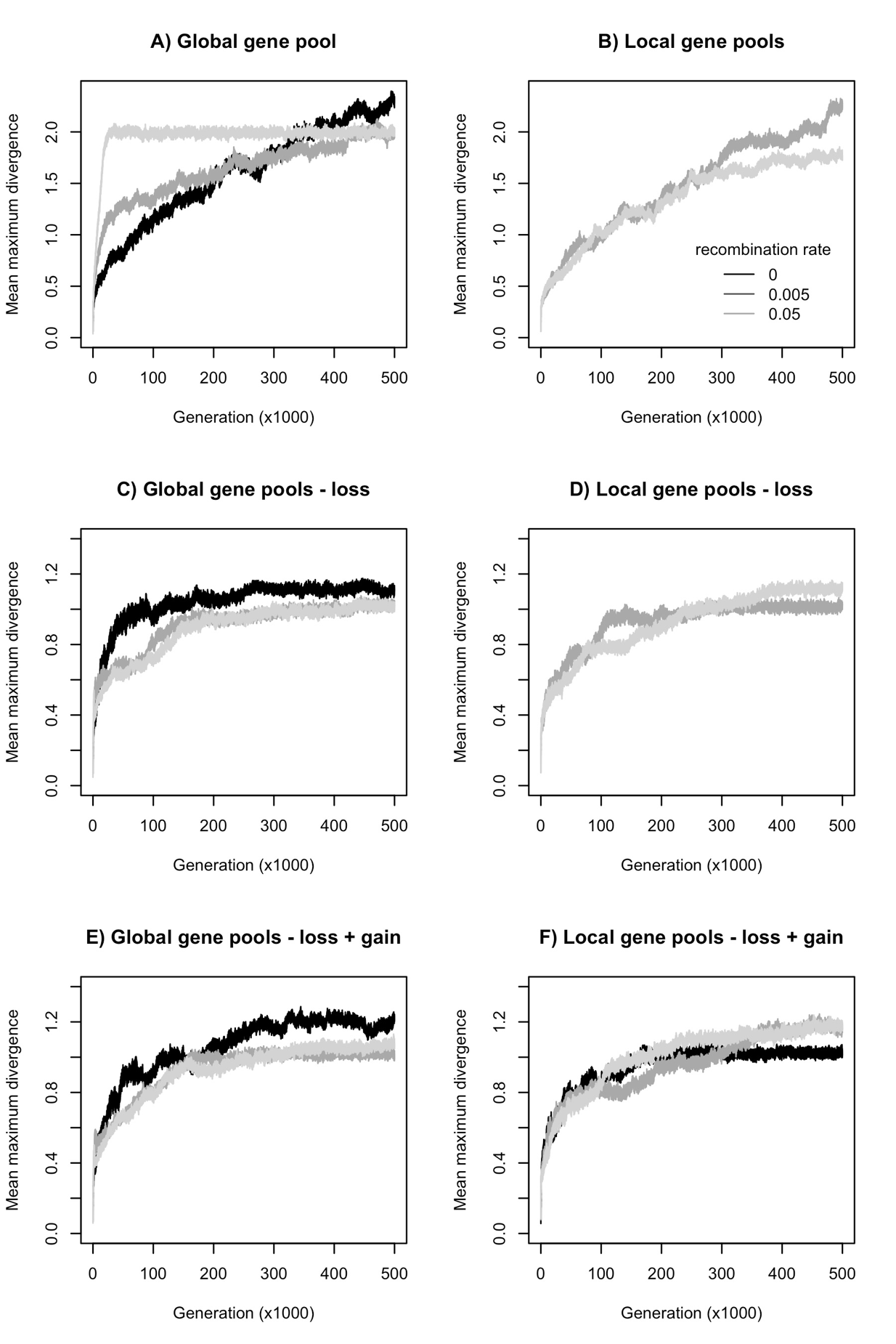


**FIGURE S5**

### FIGURE S6


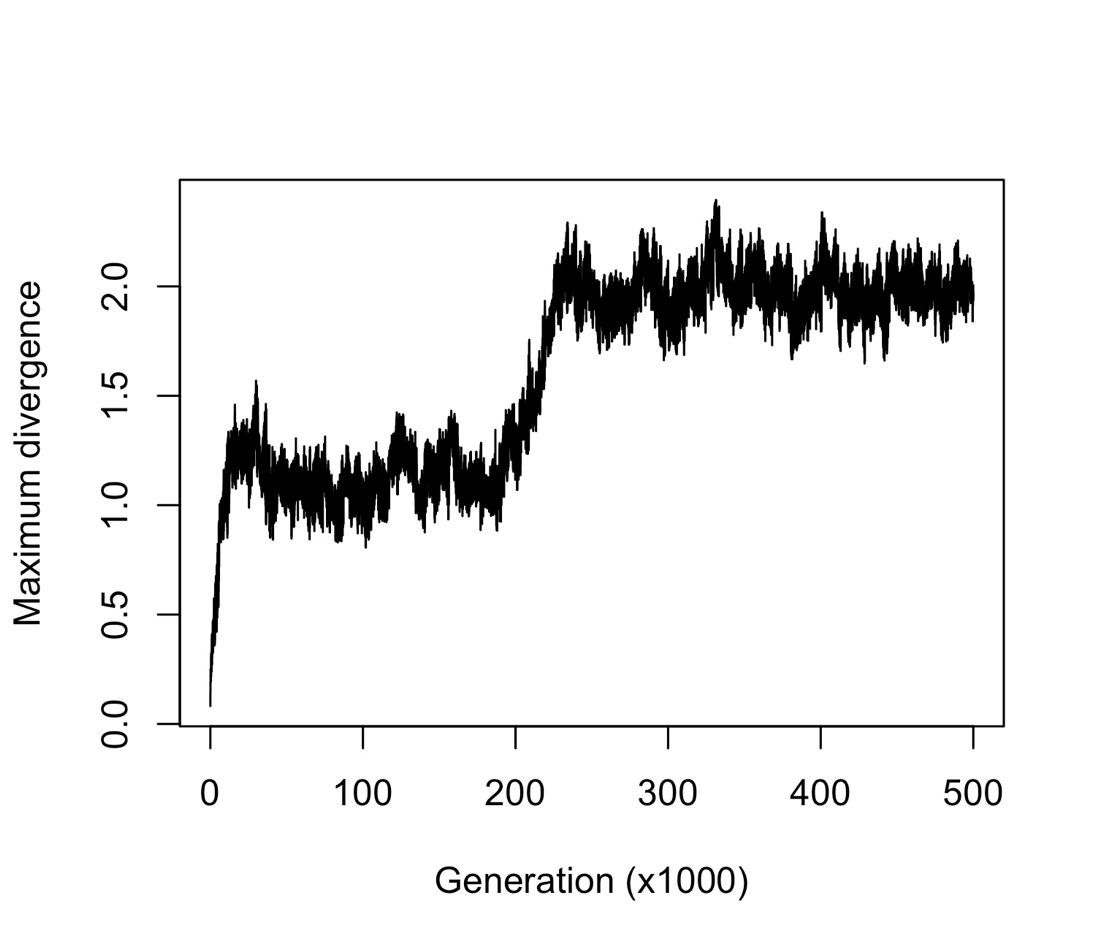


**FIGURE S7**

**FIGURE S8**
